# Animal as the Solution II: Phenotyping for Low Milk Urea Nitrogen A1PF Dairy Cows

**DOI:** 10.3390/ani15010032

**Published:** 2024-12-26

**Authors:** Fabiellen C. Pereira, Sagara Kumara, Anita Fleming, Shu Zhan Lai, Ella Wilson, Pablo Gregorini

**Affiliations:** Department of Agricultural Sciences, Faculty of Agricultural and Life Sciences, Lincoln University, Lincoln, P.O. Box 85084, Christchurch 7647, New Zealand; sagara.kumara@lincoln.ac.nz (S.K.); anita.fleming@lincoln.ac.nz (A.F.); l.laishuzhan@lincolnuni.ac.nz (S.Z.L.); pablo.gregorini@lincoln.ac.nz (P.G.)

**Keywords:** milk urea nitrogen, urinary nitrogen, dairy cows

## Abstract

The societal pressure on intensive pastoral dairying demands the search for strategies to reduce the amount of N flowing through and excreted by dairy cows. One of the strategies that is being currently explored focuses on the animal as a solution, as there are differences in N metabolism between cows even within the same herd. This work was conducted to explore such an approach in A1PF herds in New Zealand and the possibility of identifying A1PF cows that are divergent for milk urea nitrogen (MUN) concentration through phenotyping as a potential viable strategy to reduce N leaching and emissions from temperate dairy systems. Three herd tests were conducted to select a population sample of 200 cows (exhibiting the lowest 100 and highest 100 MUN concentrations). Milk samples were collected from the 200 cows during mid and late lactation to test for milk solids content and MUN. From the 200 cows, urine for urinary N concentration (UN), blood for plasma urea N, total antioxidants (TAS), and glutathione peroxidase (GPx) were collected from the 20 extremes (the lowest 10 and highest 10 MUN concentrations). Milk urea N was greater in cows selected as high-MUN cows (16.2 vs. 14.32 ± 0.23 mg/dL) and greater during late lactation (16.9 vs. 13.0 ± 0.19 mg/dL). Milk solids and fat content were 38% and 20% greater in cows selected as low-MUN cows than in high-MUN cows during mid lactation (*p* < 0.001). Low-MUN cows had lower UN than high-MUN cows during mid lactation (0.64 vs. 0.88 ± 0.11%). The N concentration in the plasma (*p* = 0.01) and Tas (*p* = 0.06) were greater during late lactation. There was a positive relationship between the MUN concentration phenotype used for selection and the MUN concentration for the trial period and MUN concentration and UN concentration during mid and late lactation (*p* < 0.001). Our results suggest that A1PF cows within a commercial herd can be phenotyped and selected for low-MUN, which may be potentially a viable strategy to reduce N losses to the environment and create healthier systems. Following genetic tracking, those cows can be bred to further promote low-MUN A1PF herds.

## Simple Summary

Society is aware of the negative environmental impacts of pastoral livestock production systems, mainly the emission of greenhouse gases and water and air pollution. A great concern is the concentration of urinary N that leaches into groundwater. However, previous research has shown that there are solutions and strategies to reduce such impacts. Some cows, for instance, have much less environmental impact, alongside producing milk with greater protein content and nutraceutical value for human consumption. If cows present different traits and, therefore, different milk yield/composition and environmental impact, we can select only cows within a herd whose traits are more beneficial to the system. In this present work, we are proposing a methodology to select cows that have lower N excretion as a strategy to reduce N losses to the environment and create healthier systems.

## 1. Introduction

Pastoral livestock-production systems are under increasing environmental, social, and consumer pressures to reduce environmental impact [1]. Pastoral dairy production systems in temperate regions like New Zealand are no exception. A major concern in New Zealand dairy farms is the amount of nitrogen fertilizer applied to maintain high productivity and high protein content pastures. The concern relates to the known inefficiency of nitrogen (N) utilization by dairy cows [2]. Approximately only 30% of the N ingested is in fact utilized to support animal production (e.g., milk, live weight gain), with the remaining N excreted, mainly (over 60%) as urinary nitrogen (UN) [2,3,4].

It has been reported that, in pasture-based dairy production systems, approximately 82% of the UN excreted is discharged onto pastures [5,6], with typically 20–30% of the N lost in this manner leached to the waterways and 2% lost as N_2_O. High levels of N in waterways have been associated with environmental pollution. Moreover, N losses to the environment can also be detrimental to human health. ‘Blue baby syndrome’ is a health problem that has been largely associated with high levels of nitrates in drinking water resulting in methemoglobinemia in infants, which can be fatal in severe cases [7]. There is also evidence of an increased risk of developing colorectal cancer [8], thyroid disease [9], and neural tube defects [10] from high levels of nitrates consumed in drinking water.

All the above confirms the need to explore strategies to reduce the amount of N flowing through and excreted by dairy cows and respond to the societal pressures regarding intensive pastoral dairying. Some potential approaches include feeding strategies, such as using different feed with lower protein content or dilution properties [11,12], and management strategies, such as limiting the intensification of farms by proposing reference values of inputs and outputs (N fertilizer and supplements, stocking rate, and productivity) [13]. Alternatively, focusing on individual animals, Marshall and Gregorini [14] propose the ‘search for’ animals that have a lower environmental impact using commercial farm-based phenotyping, as animal-based solutions are permanent, cumulative, and often more cost-effective system-based approaches [13,15,16].

Marshall and Gregorini [14] used the animal-based solution approach in New Zealand herds and were successful in identifying cows within a herd with reduced N excretion in their urine, greater frequency of urination with smaller volume, and lower N concentration [17]. Furthermore, those cows produced milk with a greater content of protein [17]. Following their approach, a commercial farm with only A1 Protein Free (A1PF) cows, also in New Zealand, participated in this current study to determine if low-MUN cows were also present in A1PF herds and if phenotyping could be used to identify these individuals. Because urea equilibrates within bodily fluids [18], the relationship between UN and MUN is linear [19], which is also correlated with urea N concentration in plasma [18]. On the other hand, the correlation between MUN and milk protein content is negative [20,21]. That means that cows with lower UN excretion will most likely allocate the dietary N from plasma into other outputs than urine, such as milk protein, potentially increasing farm profitability [17]. The heritability of MUN for dairy cows in New Zealand was calculated as 0.22 [20]. In other words, in dairy cows, 22% of the phenotypic variation for MUN is due to genetics, which indicates that cows can be selected and bred for low-MUN. Therefore, MUN could be a useful phenotyping tool for improving N use efficiency [22] and minimizing the environmental impacts of pastoral dairying in temperate regions by creating low-MUN herds.

Phenotypes are a product of genotype and development within a particular environment. Therefore, with variable environments and a constant phenotype over time, breeding A1PF low-MUN phenotypes could emerge as a genetic selection strategy tool to address both impacts. This work presents the identification of low-MUN A1PF cows within a large commercial A1PF herd and a detailed follow-up of those cows in terms of UN to assess the potential reduction in environmental impact.

## 2. Materials and Methods

### 2.1. Research Sites, Animals and Phenotyping

The study was conducted in two commercial A1PF herds from Align farms Ltd., Canterbury, New Zealand (44°00′58.8″ South 171°25′34.4″ East) from September 2023 to March 2024. As an on-farm trial, specific measurements such as individual milk yield, dry matter intake, and urine volume were not available, so the study was designed according to the possible measurements to be taken.

The two herds totaled 1600 Holstein–Friesian cows (liveweight of 520 ± 50 kg and 7.35 ± 0.85% milk solids (fat + protein concentration) per day), calving between mid-July and mid-August (2023) and phenotyped for MUN excretion (three consecutive milk samplings) in October (i.e., early lactation). A population sample of 200 cows was then chosen based on MUN values (the lowest 100 and highest 100 MUN). These two MUN contrasting groups of cows were then evaluated over lactation at two points—mid (around early February) and late lactation (around the end of March) for milk composition. From then, we selected the 20 extreme cows (with the lowest 10 and highest 10 MUN concentrations), which were also evaluated for blood and urine urea concentration as well as total antioxidant status, as described below (more information about the selected cows can be found in the Supplementary Materials). All the selected cows were allocated to the same herd and grazed on perennial ryegrass (*Lolium perenne* L.)-based swards during sampling times at mid and late lactation.

The number of animals used was based on a power analysis conducted for finding statistical differences in urinary urea nitrogen (UUN) between high- and low-MUN breeding value cows based on Marshall, Beck [23] (*n* = 8, SD = 0.38). With the desired power of 95% and a significance level of 0.05, an expected difference of 1.02 could be detected using seven experimental units for each treatment. Considering the same study (n = 8, SD = 0.19), with the desired power of 95% and a significance level of 0.05, 82 experimental units would be needed to find an expected difference of 0.17 between high- and low-MUN breeding value cows for total milk solids production.

### 2.2. Measurements

Herbage samples were collected by hand-plucking during pre-grazing for DM-based botanical and chemical composition. For chemical composition, a subsample was freeze-dried, ground to pass through a 1 mm sieve (ZM200 Retsch), and analyzed by using near-infrared spectrophotometry (NIRS, Model: FOSS NIRSystems 5000, Silver Spring, MD, USA). Chemical composition values used for NIRs calibration were derived before sample analysis for DM (AOAC, 1990; method 930.15), NDF [24], ADF [25] (method 973.18), WSC [26], DOMD, DMD [27], CP by combustion (Variomax CN Analyser Elementar, Marlton, NJ, USA), and OM by digestibility [27]. NIRs calibration equations all had R2 values greater than 0.90 and were within the calibration range.

Milk samples were analyzed for urea, protein, fat, and lactose concentrations and somatic cell count (MilkTest New Zealand, Hamilton, New Zealand) using a CombiFoss machine (Foss Electric, Hillerød, Denmark).

Urine samples were obtained by stimulating the vulva to induce urination and immediately acidified with sulphuric acid to prevent ammonia (NH_3_) volatilization. The urea UN concentration was determined using a commercial enzymatic kinetic technique in an automatic clinical analyzer (Randox Daytona; Crumlin, UK). Urea UN concentration was the target trait measured, as N intake has little effect on the urinary non-urea fraction of total urinary N, whereas N intake explains 92 to 99% of the additional N excretion in urine in the form of urea [28].

Blood samples were collected via venipuncture of the coccygeal vein into 10 mL lithium heparinized evacuated tubes (Greiner Bio-One International GmbH, Kremsmunster, Austria). A subsample of the whole blood was taken for further glutathione peroxidase (GPx) analysis using an enzymatic method, according to the manufacture’s specifications (RANSEL, Cat. No. RS504; Crumlin, UK). The remaining blood was centrifuged at 3000× *g* for 10 min at 4 °C using a Megafuge 1.0R (Heraeus Holding GmbH, Hanau, Germany) to obtain plasma. Plasma urea N (PUN) was determined with an enzymatic kinetic technique using a clinical analyzer (Randox Daytona; Crumlin, UK). Plasma total antioxidant status (TAS; Cat. No. NX2332) was analyzed according to the Randox kit manual using the Randox Rx Daytona; Crumlin, UK).

### 2.3. Statistical Analysis

Statistical analyses were performed in R [29] using the lme4 package [30]. The effects of cows differing in MUN and sampling period (mid or late lactation) on milk solids, UN, PUN, TAS, and GPx were tested using generalized linear mixed effects models. Group (high or low) and sampling period (mid or late) were included as fixed effects, while individual cows were considered random effects. The effects of group (low and high-MUN) and sampling period, and their interaction, were determined by an analysis of deviance table, using a type II Wald Chi-square test. The MUN phenotype of cows was modeled on measured variables by including it and the sampling period as fixed terms, with days of pregnancy, days in lactation nested with sampling period, and the age of the cow as covariates. Model assumptions were adjusted graphically for normal distribution and homoscedasticity of the residuals. Data were logarithmically transformed when they did not respect model assumptions. Statistical significance was determined at *p* ≤ 0.05 and tendencies are discussed at 0.05 < *p* ≤ 0.10.

## 3. Results

The botanical and chemical composition of the diet is presented in Table 1.

The difference between the lowest and the highest cow regarding MUN phenotype during early lactation was 2.0 to 19.8 mg/dL.

Milk urea N was greater (Table 2) in the group of high-MUN cows (16.2 vs. 14.32 ± 0.231 mg/dL) and greater during late lactation (16.9 vs. 13.0 ± 0.19 mg/dL). There was no difference between high and low-MUN cows for milk protein or lactose content. Milk fat content was on average 20% greater in low-MUN cows than their high-MUN counterparts during mid lactation.

There was an interaction between the group and sampling period for UN (Table 3). Low-MUN cows presented a lower UN than high-MUN cows during mid lactation (0.64 vs. 0.88 ± 0.11%). Plasma urea N was numerically lower in low-MUN cows compared to high-MUN cows, although there was no statistical difference (*p* = 0.15). Plasma urea N (PUN) (*p* = 0.01) and TAS (*p* = 0.06) were both greater during late lactation.

The protein content of milk was greater during late lactation than early lactation (4.6 vs. 4.3 ± 0.38%; *p* = 0.02), and the lactose content was greater during mid lactation than late lactation (5.06 vs. 4.78 ± 0.18, *p* < 0.0001). There was no effect of MUN on protein (*p* = 0.23) or lactose content (*p* = 0.17). The percentage of fat in milk increased in late lactation (5.3 ±1.26%) compared with mid lactation (3.1 ± 1.86%, *p* < 0.01). The fat content of milk decreased by 0.03 ± 0.01% per unit increase in MUN (*p* < 0.01) across both stages of lactation.

**Figure 1:**
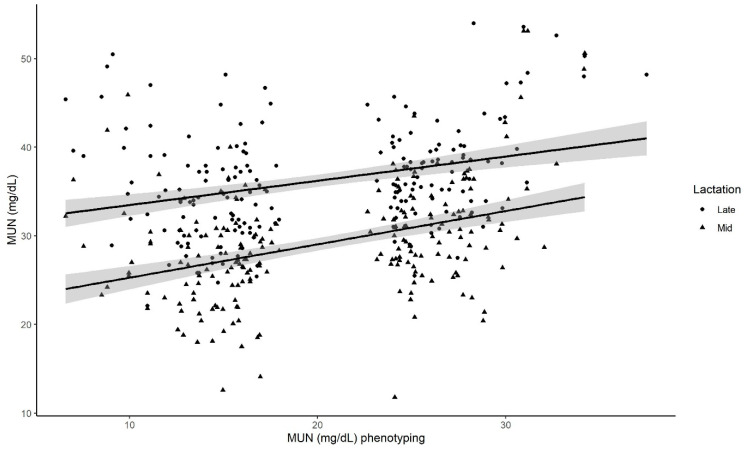
The milk urea N (MUN) values from cows during sampling points at mid and late lactation are affected by the cow MUN value at the phenotyping. For every one-unit increase in MUN at the phenotyping, there was a subsequent 0.35 mg increase in N from urea per dL of milk (*p* < 0.0001). The sampling period affected the intercept of the regression line (*p* < 0.0001). The regression model’s adjusted R^2^ = 0.32.

Milk urea N increased by 23.8% in late as compared with mid lactation (13.6 to 16.8 mg/dL, *p* < 0. 01). The values of MU during mid and late lactation were positively correlated with cows’ MU values during the herd phenotyping, increasing 0.35 ± 0.04 mg/dL per unit increase in MUN at the phenotyping (*p* < 0.01, Figure 1). Similar results were found for the 20 extremes of low- and high-N cows. Milk urea N increased by 22% in late as compared with mid lactation (20.4 to 15.8 mg/dL, *p* < 0.01), and there was a 0.36 ± 0.19 mg/dL increase in MUN during mid and late lactation per unit increase in MUN at the phenotyping (*p* < 0.01).

Urea UN concentration was positively correlated with cows’ MUN values during the herd phenotyping, increasing in 0.07 g/L urine N from urea per unit increase in MUN at the phenotyping (*p* = 0.03) across both stages of lactation. There was a tendency for sampling periods to affect the intercept of the regression line for UN. The intercept was 1.63 for mid lactation and 2.75 g UN from urea per L of urine for late lactation (*p* = 0.09).

The intercept of the regression line for PUN changed from mid to late lactation from 11.3 to 4.7 g blood N from urea per L of blood (*p* = 0.03). However, there was no effect of MUN at the phenotyping on PUN during mid and late lactation (*p* = 0.28). There was no effect of MUN on TAS (*p* = 0.16) and GPx (*p* = 0.51) concentration in blood, but there was a tendency for a reduction of 15% TAS in mid lactation as compared to late lactation (*p* = 0.06).

## 4. Discussion

A herd of 1600 A1PF dairy cows was phenotyped for MUN and the 20 extreme cows (the 10 highest and the 10 lowest cows for MUN concentration) were selected to investigate the relationship between MUN and UN, PUN, milk solids, and other blood parameters over two stages during lactation. Our results indicate a positive linear relationship between MUN and UN, and MUN and milk fat content, during mid and late lactation stages. It is important to note that this is an on-farm trial, so not all animal measurements were available. The interpretation of the results was speculated according to the measurements taken.

Based on this and previous reports [17], considering the heritability of MUN as 0.22 for dairy cows in New Zealand, a positive relationship between MUN and UN in A1PF cows strongly suggests that these cows can be phenotyped, selected, and bred for low-MUN as a strategy to reduce N source pollution to the environment. Between 2 and 3% of the UN deposited onto pasture is converted to N_2_O [4], UN being the greater source of N_2_O in pastoral livestock production systems and therefore representing a significant detrimental effect to the environment [31]. In general, the UN excretion is in the order of 13 to 16 g per mg of MUN/dl per day. In our study, MUN varied from 5.50 to 24.78 mg/dL during mid lactation and 10.31 to 25.2 mg/dL during late lactation. This would represent a difference of 75 and 60% in UN excretion during mid and late lactation, respectively, between the lowest and the highest MUN cow. Furthermore, based on MUN values, UN increases 0.07 g/L per unit increase in MUN across both stages of lactation. Such a reduction indicates that high-MUN cows would have greater UN as compared to low-MUN cows; therefore, phenotyping, selecting and then breeding cows for low-MUN is a potential strategy for N_2_O production in temperate pastoral livestock, but further research is required to verify the accuracy of this technique.

Marshall et al. (2020) reported an average of 2.85 L per urine event, covering an area of 0.57 m^2^ [32]. To illustrate our findings and the magnitude of the extremes based on which potential selection for animals may occur, and assuming that the cows used in our study would have the same urine volume per event and the same urination frequency based on models proposed by Haynes and Williams [33] and Selbie, Buckthought [4], we estimated the difference in UN deposited onto pasture between the lowest and the highest N cow:

$$UN\;rate\;kg/ha=\frac{UN\;g/L*Vol\;L}{Area\;per\;urination\;event\;m^{2}}\times 10$$

The expected differential load of UN per hectare (at urine patch level) between the cow with the lowest and the highest MUN across both stages of lactation was 190 kg, which would represent a 45% reduction in UN loading per ha. Since there is an exponential relationship between N load onto pastures and N leaching [34,35], using the empirical model of Di and Cameron [34], we calculated a difference of 41.37 kg NO_3_ N leached per ha between the cow with the lowest and the highest MUN. Such a difference constitutes a tremendous positive impact—i.e., a massive reduction in the negative impact of pastoral dairying to the environment—that would allow us to speculate that, if all the cows in New Zealand could be phenotyped and then selected for low MUN at the level found in our study, future dairy impacts on both water and climate could be significantly reduced.

$$y=16.7+0.17x+0.000071x^{2}$$

where *y* = total NO_3_ N leached (kg/ha) and *x* = UN load onto pasture (kg/ha).

Considering that 2% of the urine deposited onto pasture is transformed to N_2_O [36,37] and that N_2_O has a global warming potential 273 times the one of CO_2_, the lowest and the highest MUN cows would, therefore, contribute to 1201.2 and 2238.6 kg of CO_2_eq, respectively. When considering milk solids and pollution intensity in terms of N_2_O, at the same level of milk production, the lowest cow would emit 12.6 kg of CO_2eq_ per kg of milk solids, while the highest cow would emit 27.36 kg of CO_2eq_ per kg of milk solids produced. The reduction in both N_2_O and N leaching to waterways is one of the main societal environmental concerns to be alleviated in temperate pastoral dairy production systems [4,38], since, as was mentioned before, such discharges of N to the environment not only affect out planetary health but our human health, in terms of cancer, blue babies, and more.

Low-MUN cows that exhibited lower UN excretion would also be expected to exhibit lower N in blood, as they would need to partition N from the diet away from urine to other N pools [20,39]. Blood urea is highly correlated with MUN and UN excretion due to urea synthesis and use [19,40]. The urea comes from the ammonia produced in the rumen from the microbial degradation of protein, synthesized by the liver, and released in the blood to either be filtered by the kidneys and excreted in the urine, diffused into milk, or go back to the gastrointestinal tract for microbial synthesis [41,42,43]. This means that animals with high-MUN and PUN are less efficient at recycling urea to the gastrointestinal tract, and both parameters are correlated [41,44]. In our study, though, MUN and UN were not correlated to PUN, with no effect of MUN on milk protein content. This could be explained by delayed blood sampling as compared to milk sampling. The lack of correlation could also indicate a divergence in N partition towards muscles or faeces [20]. Greater N excretion through faeces is favorable to the environment, as faecal N is more organically stable and less volatile than urea in urine, readily converted to NH_3_ [45]. Nitrogen partition to faeces could indicate that the animals are more efficient in using dietary N, if milk protein content is increased, or it could indicate a reduction in protein degradation in the rumen [45]. Either way, N partition away from urine to faeces is an environmental advantage. Since the protein content in milk and PUN was not different between cows selected for high MUN and those selected for low MUN, that could reflect differences in faeces due to the difference in MUN and UN. Further research is needed regarding N partition to faeces in cattle diverging in MUN phenotype.

Milk protein and fat increased during late lactation as compared to mid lactation, while milk lactose content decreased, which agrees with previous research [17,46,47]. Greater protein content is also a reflection of diet, as during late lactation the protein content of the diet was greater than during mid lactation. Greater protein content in the herbage also contributes to explaining a greater MUN during late lactation. Low-MUN cows, in addition to having lower MUN and lower UN deposited onto pasture, also had greater milk fat content than high-MUN cows, which may add to greater milk solid production.

In summary, phenotype equals genotype × environment; consequently, if the phenotype remains constant at variable environment, it can indicate a strong influence of genotype on such features. In other words, breeding low-MUN phenotypes emerge as a potential genetic selection strategy tool, including in A1PF herds, to improve N use efficiency and reduce environmental impacts from temperate pastoral livestock production systems. A one-unit increase in MUN during the phenotyping elicited an increase of 0.35 mg/dL in MUN during mid and late lactation. This indicates that cows are divergent with MUN values and that they can be phenotyped and selected for low-MUN. As MUN is a hereditable feature [48], phenotyping cows for low-MUN is part of the solution to reduce N losses to the environment in temperate pastoral livestock production systems in the short and long term. Nevertheless, this study was conducted in one herd and one lactation, so further studies exploring other herds and other environments are needed to confirm the phenotyping approach. In addition, since the study was conducted on a commercial farm, some important measurements such as urine volume, dry matter intake, and milk yield were unavailable. The conclusions of this study were speculated based on the measurements taken, but further studies exploring other variables would enhance the interpretation of the results. Furthermore, the genotyping of the selected cows could contribute to verifying the accuracy and applicability of using phenotyping as an animal-based strategy to reduce N losses in temperate pastoral dairy systems.

## 5. Conclusions

The divergence between animals in terms of MUN and UN strongly suggests that dairy cows, including those in A1PF herds, can be phenotyped and selected for low MUN. Milk urea N is a hereditable feature (0.22) related to different genes, and the genotyping of the selected animals could verify the accuracy and applicability of such a strategy. However, since genotyping is still not an easy and affordable method for farmers, phenotyping would be a provisory strategy to potentially create low-MUN herds, by selecting MUN cows and breeding them with low-MUN breeding value bulls. The phenotyping was performed during herd tests for milk composition, which are already a common practice in New Zealand dairy farms. If further studies in other herds and environments confirm the use of phenotyping to select low-MUN cows, phenotyping could be a practical technique that farmers could use as a viable strategy to reduce N losses to the environment in temperate pastoral livestock production systems in the short and long term.

## Figures and Tables

**Table 1:** Botanical and chemical composition of the herbage diet grazed by high- and low-MUN cows during mid and late lactation.

	Mid Lactation	Late Lactation
Italian ryegrass (%)	1.87	0
Perennial ryegrass (%)	66.72	68.56
White clover (%)	12.51	6.88
Weed (%)	6.81	1.66
Dandelion (%)	10.50	6.96
Chicory (%)	0	3.56
Plantain (%)	0	3.06
Dead material (%)	1.59	9.29
DM %	20.76	28.48
OM, % DM	91.18	90.77
CP, % DM	18.55	20.7
NDF, % DM	39.71	36.55
ADF, % DM	23.30	21.62
WSC, % DM	18.22	17.91
DMD %	79.25	81.44

Italian ryegrass: *Lolium multiflorum*; Perennial ryegrass: *Lolium perenne*; White clover: *Trifolium repens*; Dandelion: *Taraxacum* spp.; Chicory: *Cichorium intybus*; Plantain: *Plantago lanceolata* L. DM, dry matter; OM, organic matter; CP, crude protein; NDF, neutral detergent fiber; ADF, acid detergent fiber; WSC, water-soluble carbohydrates; DMD, dry matter digestibility.

**Table 2:** Mean and standard deviation of milk urea N and milk solids from cows differing in MUN (milk urea N breeding value) during mid and late lactation (High = high N and Low = low N); n = 200.

	Mid Lactation			Late Lactation			*p*-Value		
	High	Low	SD	High	Low	SD	Group	Lactation	Inter.
MUN mg/dL	14.68	12.56	2.86	17.75	16.11	2.73	≤0.001	≤0.001	0.19
Milk protein %	4.25	4.29	0.32	4.58	4.59	0.38	0.37	≤0.001	0.93
Milk fat %	2.77	3.47	1.77	5.36	5.31	1.26	0.04	≤0.001	0.01
Lactose %	5.05	5.07	0.20	4.77	4.79	0.18	0.47	≤0.001	0.81
SSC	78 × 10^3^	31 × 10^3^		72 × 10^3^	57 × 10^3^		0.53	0.56	0.11

*p*-value lower than 0.05 means significative difference. Inter. = interaction between group and sampling period.

**Table 3:** Mean and standard deviation of urinary nitrogen (UN %), plasma urea N (PUN %), total antioxidants (Tas mmol/L), and glutathione peroxidase (GPx U/L) from cows differing in MUN (milk urea N breeding value) during mid and late lactation (High = high N and Low = low N); n = 20.

	Mid Lactation			Late Lactation			*p*-Value		
	High	Low	SD	High	Low	SD	Group	Lactation	Inter.
UN %	0.88	0.64	0.11	0.67	0.64	0.22	0.06	0.01	0.06
PUN (mmol/L)	7.97	7.56	1.31	8.93	7.96	0.77	0.15	0.01	0.35
Tas (mmol/L)	0.74	0.79	0.17	0.82	0.93	0.15	0.16	0.11	0.82
GPx (U/L)	41,161.95	47,190.86	11,990.9	44,194.23	47,660	12,025.2	0.51	0.82	0.86

*p*-value lower than 0.05 means significative difference. Inter. = interaction between group and stage of lactation.

## Data Availability

The raw data supporting the conclusions of this article will be made available by the authors on request.

## Supplementary Materials

The following supporting information can be downloaded at: https://www.mdpi.com/article/10.3390/ani15010032/s1.
